# Repeated measures discriminant analysis using multivariate generalized estimation equations

**DOI:** 10.1177/09622802211032705

**Published:** 2021-12-13

**Authors:** Anita Brobbey, Samuel Wiebe, Alberto Nettel-Aguirre, Colin Bruce Josephson, Tyler Williamson, Lisa M Lix, Tolulope T. Sajobi

**Affiliations:** 1 Department of Community Health Sciences, 2129 University of Calgary, University of Calgary, Calgary, Canada; 2 Department of Clinical Neurosciences, 2129 University of Calgary, University of Calgary, Calgary, Canada; 3 Centre for Health and Social Analytics, 8691 University of Wollongong, National Institute for Applied Statistics Research Australia, University of Wollongong, Wollongong, Australia; 4 Department of Community Health Sciences, University of Manitoba, Winnipeg, Canada

**Keywords:** Discriminant analysis, multivariate repeated measures data, generalized estimating equation, multivariate non-normal distribution, classification

## Abstract

Discriminant analysis procedures that assume parsimonious covariance and/or means structures have been proposed for distinguishing between two or more populations in multivariate repeated measures designs. However, these procedures rely on the assumptions of multivariate normality which is not tenable in multivariate repeated measures designs which are characterized by binary, ordinal, or mixed types of response distributions. This study investigates the accuracy of repeated measures discriminant analysis (RMDA) based on the multivariate generalized estimating equations (GEE) framework for classification in multivariate repeated measures designs with the same or different types of responses repeatedly measured over time. Monte Carlo methods were used to compare the accuracy of RMDA procedures based on GEE, and RMDA based on maximum likelihood estimators (MLE) under diverse simulation conditions, which included number of repeated measure occasions, number of responses, sample size, correlation structures, and type of response distribution. RMDA based on GEE exhibited higher average classification accuracy than RMDA based on MLE especially in multivariate non-normal distributions. Three repeatedly measured responses namely severity of epilepsy, current number of anti-epileptic drugs, and parent-reported quality of life in children with epilepsy were used to demonstrate the application of these procedures.

## 1 Introduction

Discriminant analysis (DA) is commonly used to classify an individual into one of two (or more) populations on the basis of correlated response measures. In more recent years, relevant work has been done in capturing the longitudinal nature of clinical data and using it for classiﬁcation via discriminant analysis.^1–8^ These research studies include DA extensions to repeated measures data with multiple response. Utilizing the correlation structure across responses with a multivariate model could increase the classification accuracy.^9^ Classical DA does not model the covariance structure, and thus the information regarding the possible structure in the covariance for repeated measurements taken on the same individual and between responses is lost.^10–13^ Moreover, classical DA is based on multivariate normality assumption to guarantee an optimal solution. Equal covariance structures are assumed in the groups^10^ for linear discriminant analysis (LDA), while quadratic discriminant analysis (QDA) allows for unequal covariance structures between the groups.^11–13^

Most DA methodologies in multivariate repeated measures data are based on mixed effects model. Multivariate linear and non-linear mixed effects models that assume unstructured^1,14^ and parsimonious structure^7,9,15,16^ for the variance-covariance matrix have been introduced. For instance, several continuous markers and a multivariate linear mixed effects model were used to evaluate a prognosis of primary biliary cirrhosis patients^14^ and non-linear mixed effects model to distinguish between women with and without pregnancy abnormalities.^15^ Similarly, three continuous markers were used to classify patients suffering from prostate cancer.^7^ Generalized linear mixed effects models have been extended in multivariate repeated measures studies for different type of responses (continuous, counts, and binary).^1,3,17^ Most mixed effects model DA assume that the random effects follow a multivariate normal distribution. Moreover, the dimension of random-effects quickly increases as more responses and more measurements occasions are added to the model, increasing the computational burden and instability.^1,8,14^ In addition, it is difficult to evaluate the marginal likelihood of jointly generalized linear mixed effects models when the response is non-normal.

Contrary to mixed effects models approaches, some researchers have utilized the generalized estimating equations (GEE) based on multiple marginal models of multiple responses. To avoid the specification of the full likelihood function especially for discrete data, GEE^18^ is a suitable approach for parameter estimation for repeated measures data without full specification of the likelihood. Specifically, GEEs directly specify a marginal mean model for each response and induce the correlation between measurements of responses through a working correlation matrix. GEEs offer a computationally non-intensive parameter estimation algorithm and the resulting parameter estimates have population-averaged interpretation. A joint modeling of multiple response variables is based on straightforward extension of univariate GEEs with correlation structure across responses which provides separate set of regression parameters for each response variable.^19,20^ GEEs are less sensitive to covariance misspecification compared to mixed effects models.^18,21^

This study examines the accuracy of discriminant analysis based on multivariate GEE framework for classification in multivariate repeated measures designs with same/different types of responses. The article is organized as follows. In section 2, we describe the GEEs framework for multivariate repeated measures data. The proposed approach, the extension of the GEE framework to discriminant analysis, is presented in section 3. In section 4, we summarize the results of a Monte Carlo simulation study to assess the accuracy of the proposed repeated measures discriminant analysis based on GEE RMDA approach under diverse simulation scenarios. Data from a multivariate longitudinal study of children with epilepsy were used to demonstrate the application of these procedures in section 5. Finally, a discussion of the key findings from the study and its implications are described in section 6.

## 2 GEE for multivariate repeated measures data

Suppose we have a random sample of *N* individuals. For each individual *i *=* *1, …, *N*, let $y_{i}={\;(y_{i1}^{\prime },y_{i2}^{\prime },\ldots ,y_{iQ}^{\prime })}^{\prime }$ be a PQ x 1 vector of *Q* correlated responses that are each repeatedly measured at *P* occasions, and $X_{i}={I_{Q}⊗\;X}_{i*}\;$ is a corresponding KQ × PQ block diagonal covariate matrix, where $X_{i*}=(X_{i1},\;X_{i2},\;\ldots X_{ik},\ldots ,\;X_{iK})$ is a  K x P matrix of covariates, $I_{Q}$ is an Q x Q identity matrix, and ⊗ is the Kronecker product symbol. For the analysis of multivariate correlated data, the marginal mean vector is associated with the covariates through a generalized linear model (GLM) as follows

(1) $$\mu_{\mathrm{ipq}}=E\left(y_{\mathrm{ipq}}|X_{ip}\right)=f(X_{ip}^{\prime }\beta_{q}),$$

where, f(·) is the inverse response-specific link function, $\beta_{q}={\left(\beta_{q1},\;\beta_{q2},\;\ldots \beta_{qk},\ldots ,\;\beta_{qK}\right)}^{\prime }$ is the *K × *1 dimensional vector of the *q*th response regression coefficients, and $X_{ip}$ is the corresponding covariate at time *p* for the *i*th individual. The KQ-dimensional parameter vector of $\beta ={\left(\beta_{1}^{'},\beta_{2}^{'},\ldots ,\ldots ,\beta_{Q}^{'}\right)}^{'}$ and the marginal model in equation (1) is represented by $\mu_{i}=E\left(y_{i}|X_{i}\right)=f\left(X_{i}^{\prime }\beta \right).$ In the quasi-likelihood framework with repeated measures responses, the regression coefficients in β can be estimated by solving the generalized estimating equations (GEEs)

(2) $$U\left(\beta \right)=\sum_{i=1}^{N}D_{i}^{\prime }\Omega_{i}^{-1}\left(y_{i}-\mu_{i}\right)=0$$

where $D_{i}=\frac{\partial \mu_{i}}{\partial \beta }$ is the block diagonal matrix of derivatives mean with respect to the regression parameters, $\mu_{i}$ is the marginal mean vector, and $\Omega_{i}$ is the *PQ* × *PQ* working covariance matrix.

The *PQ* × *PQ* marginal covariance matrix is

(3) $$\Omega_{i}=\phi \Sigma_{i},$$

where ϕ is a scale parameter that can be known or estimated and $\Sigma_{i}$ is an *PQ* × *PQ* working covariance matrix, which results in a total of *PQ* (*PQ *+* *1)/2 unknown parameters to be estimated^22,23^ which may not always be feasible (i.e. when *PQ* is close to *N*). To reduce the dimension of the unknown parameters of the covariance matrix, a parsimonious structure is sometimes used, such as a Kronecker product covariance structure

(4) $$\Sigma_{i}=A_{i}^{1/2}R(\alpha )⊗R(\rho ))A_{i}^{1/2}$$

where $A_{i}$ is an *PQ × PQ* block diagonal matrix, which contains the marginal variance of responses on the main diagonals, **R** (α)  is a *Q× Q* working correlation matrix of the responses with the parameter vector α , and **R** (ρ)  is the working correlation matrix for a given response at different time points with the parameter ρ. This structure reduces the number of covariance parameters to be estimated.^23–27^ Consequently, **R** (α)  and **R** (ρ) denote between-response correlation matrix and within-response correlation matrix, respectively. Further, assuming a structured working correlation, such as exchangeable (EX), first-order autoregressive (AR1), or unstructured (UN), for **R** (α)  and exchangeable (EX) or unstructured (UN) structures for **R** (ρ) , can lead to an even more parsimonious model.^22,28,29^ The parsimonious structure provides flexible model for covariance, particularly when sample size is small.^22,28,29^ Inferences of interest are easily influenced by the correlation structure’s assumptions, and unstructured correlation structure might cause convergence problems as the number of parameters to be estimated grows rapidly.^30^ Specifically,  Uβ=0 are solved with a Fisher-Scoring algorithm such that

(5) $$\hat{\beta }=\tilde{\beta }+{\left(\sum_{i=1}^{N}{\tilde{D}}_{i}^{\prime }{\tilde{\Omega }}_{i}^{-1}{\tilde{D}}_{i}\right)}^{-1}\left(\sum_{i=1}^{N}{\tilde{D}}_{i}^{\prime }{\tilde{\Omega }}_{i}^{-1}\left(y_{i}-\mu_{i}\right)\right)\;$$

Under mild regularity conditions, the parameter estimates are consistent and asymptotically normally distributed even when the “working” correlation structure of responses is misspecified, and the variance-covariance matrix can be estimated using a robust “sandwich” variance estimator.^31^ The asymptotic covariance matrix of the non-vanishing (non-zero) component of $\hat{\beta }$ via the sandwich estimator formula is^31,32^

(6) $$\hat{\mathrm{cov}}\left(\hat{\beta }\right)={\left(\sum_{i=1}^{N}{\hat{D}}_{i}^{\prime }{\hat{\Omega }}_{i}^{-1}{\hat{D}}_{i}\right)}^{-1}{\hat{M}}_{*}\;{\left(\sum_{i=1}^{N}{\hat{D}}_{i}^{\prime }{\hat{\Omega }}_{i}^{-1}{\hat{D}}_{i}\right)}^{-1}\;,$$

with

(7) $${\hat{M}}_{*}=\sum_{i=1}^{N}{\hat{D}}_{i}^{\prime }{\hat{\Omega }}_{i}^{-1}\hat{\mathrm{cov}}\left(y_{i}\right){\hat{\Omega }}_{i}^{-1}{\hat{D}}_{i}$$

and $\hat{\mathrm{cov}}\left(y_{i}\right)=\left(y_{i}-{\hat{\mu }}_{i}\right){\left(y_{i}-{\hat{\mu }}_{i}\right)}^{\prime }$ is an estimator of the true variance-covariance matrix of $y_{i}.$ ^18,31^ Note that if $\Omega_{i}\;$ is correctly specified, $\Omega_{i}=\mathrm{cov}(y_{i}).$ ^33,34^ Moreover, GEE requires the correct specification of marginal mean and variance as well as the link function, which connects the covariates of interest and the marginal means.

## 3 GEE extension to multivariate repeated measures discriminant analysis

Following the GEE notation, we assume that the *i*th individual in the *j*th population ( j= 1,2) with multivariate repeated responses $y_{ji},\;$ has a marginal mean $\mu_{\mathrm{ji}}$ , and variance covariance matrix $\Omega_{ji}$ assumed to be *PQ* × *PQ* positive definite. Analogously, with estimations of ${\hat{\mu }}_{ji}=f(X_{ji}{\hat{\beta }}_{j})$ and the variance covariance matrix ${\hat{\Omega }}_{ji}$ from the GEE model in population  j using a pre-defined structure, the homoscedastic model is obtained when the variance components are homogeneous, that is, ${\;\Omega }_{1}={\;\Omega }_{2}=\Omega ,\;$ the pooled covariance matrix. Based on LDA, a randomly selected *i*th individual with multiple response vector $y_{i}$ is classified in the first group, if

(8) $${\left(y_{i}-\frac{{\hat{\mu }}_{1}+{\hat{\mu }}_{2}}{2}\right)}^{\prime }\;{\hat{\Omega }}^{-1}({\hat{\mu }}_{1}-{\hat{\mu }}_{2})>\log\;\frac{{\hat{\pi }}_{2}}{{\hat{\pi }}_{1}}$$

where ${\hat{\mu }}_{j}\;$ and ${\hat{\Omega }}_{j}^{-1}$ are the GEE estimates from equations (1) and (2), $\;{\hat{\pi }}_{1}\;$ and ${\hat{\pi }}_{2}$ are the *a priori* probabilities that observations belong to populations 1 and 2. Otherwise, the individual is classified into the second group. For QDA (i.e. ${\;\Omega }_{1}\neq {\;\Omega }_{2})$ , the *i*th subject with multiple response vector $y_{i}$ is classified in the first group, if

(9) $${\left(y_{i}-{\hat{\mu }}_{2}\right)}^{\prime }{\hat{\Omega }}_{2}^{-1}\left(y_{i}-{\hat{\mu }}_{2}\right)-{\left(y_{i}-{\hat{\mu }}_{1}\right)}^{\prime }{\hat{\Omega }}_{1}^{-1}\left(y_{i}-{\hat{\mu }}_{1}\right)\;>\log\;\left|\frac{{\;\hat{\Omega }}_{1}}{{\;\hat{\Omega }}_{2}}\right|+2\log\;\frac{{\hat{\pi }}_{2}}{{\hat{\pi }}_{1}},$$

otherwise, it is classified into the second group.

## 4 Simulation study

A Monte Carlo simulation study was conducted to examine the accuracy of linear and quadratic GEE discriminant analysis procedures that assume Kronecker product structured covariances compared to MLE repeated measures discriminant analysis based on structured covariances.^22,29,35^ The following conditions were investigated: (a) number of repeated measurements (P), (b) total sample size  (N) , (c) group sizes $\left(n_{1},\;n_{2}\right)$ , (d) pattern and magnitude of correlation among the repeated measurements ( ρ) , (e) mean configuration, (f) covariance heterogeneity, and (g) population distribution. All procedures were investigated for two independent groups. The number of repeated occasions/time points was set at P= 3 and 5, and number of responses was set at Q= 3 and 5. Previous studies about DA procedures for multivariate repeated measures data have considered *P* ranging from 3 to 10, an increase in classification accuracies were quite significant when P increased from three to five.^36,37^ Total sample sizes of N=80 , 140 and 200 were investigated. This is consistent with previous simulation studies that examined the accuracy of DA for multivariate repeated measures data between 60 and 500. Moreover, consistent with previous studies that examined the impact of equal and unequal group sizes,^36–39^ we investigated conditions of N=80 , $(n_{1},\;n_{2})=(40,\;40),$ and ( 32, 48), which represent a group size ratio of 1:1 and 2:3, respectively. Similar equal and unequal group size ratios were investigated when N=140  and N=200 . Furthermore, the accuracy of DA procedures is known to be influenced by both the magnitude and pattern of within- and multivariate-response correlations.^40^ Therefore, we investigated the following within-response correlation structures: (a) Compound Symmetry with ρ=0.3 and ρ=0.7 , (b) autoregressive order 1 with ρ=0.3 and ρ=0.7 ^36,37^ for **R** (ρ) , and the between-responses correlation, **R** (α) was assumed to be unstructured (See Table 1 for more details).

**Table 1.** Configuration of unstructured between-responses correlation matrix given within-response correlation coefficient for the Monte Carlo Study.

Within-response	
Correlation 0.3	0.7
Coefficient ( ρ )	
$Q=3\;\left[\begin{array}{ccc} 1 & 0.15 & 0.30\\ 0.15 & 1 & 0.45\\ 0.30 & 0.45 & 1 \end{array}\right]$	$\left[\begin{array}{ccc} 1 & 0.65 & 0.66\\ 0.65 & 1 & 0.70\\ 0.66 & 0.70 & 1 \end{array}\right]$
$\;Q=5\;\;\left[\begin{array}{ccc} 1 & 0.28 & \begin{array}{ccc} 0.25 & 0.28 & 0.28 \end{array}\\ 0.28 & 1 & \begin{array}{ccc} 0.30 & 0.40 & 0.23 \end{array}\\ \begin{array}{c} 0.25\\ 0.28\\ 0.28 \end{array} & \begin{array}{c} 0.30\\ 0.40\\ 0.23 \end{array} & \begin{array}{ccc} \begin{array}{c} 1\\ 0.24\\ 0.24 \end{array} & \begin{array}{c} 0.24\\ 1\\ 0.37 \end{array} & \begin{array}{c} 0.24\\ 0.37\\ 1 \end{array} \end{array} \end{array}\right]$	$\left[\begin{array}{ccc} 1 & 0.70 & \begin{array}{ccc} 0.79 & 0.64 & 0.70 \end{array}\\ 0.70 & 1 & \begin{array}{ccc} 0.73 & 0.65 & 0.74 \end{array}\\ \begin{array}{c} 0.79\\ 0.64\\ 0.70 \end{array} & \begin{array}{c} 0.73\\ 0.65\\ 0.74 \end{array} & \begin{array}{ccc} \begin{array}{c} 1\\ 0.63\\ 0.62 \end{array} & \begin{array}{c} 0.63\\ 1\\ 0.62 \end{array} & \begin{array}{c} 0.62\\ 0.62\\ 1 \end{array} \end{array} \end{array}\right]$

*Q*: Number of responses.

Hence, we assumed two Kronecker correlation structures; UNAR = Unstructured between-responses and Autoregressive order-1 within-response correlation matrix, and UNCS = Unstructured between-responses and Compound symmetry within-response correlation matrix. For covariance heterogeneity, we assumed $\Omega_{1}=\Omega_{2}\;\mathrm{and}\;\Omega_{1}={3\Omega }_{2}$ .

In order to assess the performance of the discriminant function, we investigated multivariate correlated continuous response variables, count response variables, and different types of correlated responses, namely Case 1, Case 2, and Case 3, respectively. Case 1: For the correlated continuous response variables, we assumed three normal variables jointly observed for $n_{j}$ subjects, where each observed at P time points. The true marginal mean response model $\mu_{\mathrm{ipq}}$ was assumed to take the following functional form that uses an identity link function

(10) $$\mu_{\mathrm{ipq}}=\;\beta_{q1}x_{ip}+\beta_{q2}t_{ip}$$

The number of covariates , K=2 , where $x_{ip}$ was generated from an independent normal random variable  N(0,1) as a time-invariant covariate, and $t_{ip}$ denoted the time of observation as a time-varying covariate. Details of the true parameters β  for population 1 and population 2 can be found in Table 2.

**Table 2.** True parameters ( β ) for population 1 and population 2 simulated data.

Population distribution	Number of responses	Population 1	Population 2
Normal/mixed-type	3	(0.3,1,2,0.1,1,1.5)	(0.6,2,4,0.2,2,3)
	5	(0.2,1,2,1.5,1,0.4,0.7,3,1.2,0.8)	(0.4,2,4,3,2,0.8,1.4,6,2.4,1.6)
Poisson	3	(0.3,0.1,0.2,0.1,0.3,0.5)	( 0.9, 0.3, 0.6, 0.3 ,0.9, 1.5)
	5	$\left(0.3\text{,}0.1\text{,}0.4\text{,}0.1\text{,}0.45\text{,}0.6\text{,}0.2\text{,}0.15\text{,}0.3\text{,}0.4\right)$	(0.9, 0.3,1.2,0.3,1.35,1.8,0.6,0.45,0.9,1.2)

On the other hand, the marginal variance matrix of responses was assumed to have a common variance of 60. Case 2: For the multivariate count response variables, data were generated from a multivariate Poisson distribution using the log link function instead of identity link in Case 1 and log transformation of time of observation as a time-varying covariate.

(11) $${\log(\mu }_{\mathrm{ipq}})=\beta_{q1}x_{ip}+\;\beta_{q2}{\log(t}_{ip})$$

The true parameters β  for population 1 and population 2 can be found in Table 2. Case 3: For generating different types of correlated responses, one of the responses generated from case 1 (multivariate normal distribution data) was converted to Bernoulli response using the NORmal-To-Anything (NORTA) algorithm^41^ with probabilities from the logit function.

Linear and quadratic discriminant analysis rules were developed using the marginal mean and variance-covariance matrix estimated via GEE, and MLE for equal and unequal covariance matrix, respectively. The classification performance of the procedures was evaluated using the overall average classification accuracy and its corresponding standard errors.

(12) $$\mathrm{Overall}\;\mathrm{classification}\;\mathrm{accuracy}=\frac{\mathrm{correct}\;\mathrm{classifications}}{\mathrm{number}\;\mathrm{of}\;\mathrm{classifications}\;(N)}$$

All combinations of simulation conditions were investigated for each procedure and each method of estimation, resulting in a total of 194 combinations. There were 500 replications for each combination. All analyses were completed in R statistical software version 3.5.3.

### 4.1 Simulation results

Tables 3 and 4 describe the average classification accuracies and standard errors of repeated measures linear and quadratic discriminant analysis based on GEE, and MLE, respectively, by population distribution, number of repeated occasions, and number of responses. For each type of estimator, there were negligible differences (< 0.04) for linear DA UNCS and UNAR procedures. But RMDA based on GEE procedures were more accurate than RMDA based on MLE among UNCS procedures. For example: for the UNCS correlation matrix under GEE, the average accuracy for P=3 was 0.74 and P=5 , it was 0.89, while for the UNCS correlation matrix under MLE, the average accuracy for P=3 was 0.66 and Q=5 , it was 0.63 when the number of responses was five (Table 3).

**Table 3.** Overall mean accuracy (standard error) for repeated measures LDA procedures based on GEE, and MLE by population distribution, number of responses, number of measurements occasions, and correlation structure.

Population distribution	Number of responses	Number of measurements occasions	GEE		MLE	
			UNAR	UNCS	UNAR	UNCS
Normal	3	3	0.62 (0.04)	0.64 (0.04)	0.63 (0.04)	0.65 (0.04)
		5	0.73 (0.04)	0.75 (0.04)	0.69 (0.04)	0.70 (0.04)
	5	3	0.68 (0.04)	0.74 (0.04)	0.66 (0.04)	0.66 (0.04)
		5	0.83 (0.03)	0.89 (0.03)	0.82 (0.03)	0.63 (0.03)
Poisson	3	3	0.88 (0.04)	0.90 (0.03)	0.79 (0.04)	0.81 (0.04)
		5	0.97 (0.02)	0.97 (0.03)	0.84 (0.05)	0.85 (0.05)
	5	3	0.99 (0.01)	0.99 (0.01)	0.89 (0.04)	0.90 (0.04)
		5	0.99 (0.01)	0.99 (0.01)	0.95 (0.02)	0.95 (0.02)
Mixed-type	3	3	0.62 (0.04)	0.63 (0.04)	0.55 (0.04)	0.55 (0.04)
		5	0.72 (0.04)	0.74 (0.04)	0.58 (0.04)	0.58 (0.04)
	5	3	0.68 (0.04)	0.72 (0.04)	0.67 (0.04)	0.57 (0.04)
		5	0.81 (0.03)	0.87 (0.03)	0.62 (0.04)	0.62 (0.04)

UNAR: unstructured between-responses and autoregressive order 1 within-response correlation matrix; UNCS: unstructured between-responses and compound symmetry within-response correlation matrix; GEE: generalized estimating equation; MLE: maximum likelihood estimation.

**Table 4.** Overall mean accuracy (standard error) for repeated measures QDA procedures based on GEE, and MLE by population distribution, Number of responses, number of measurements occasions, and correlation structure.

Population distribution	Number of responses	Number of measurements occasions	GEE		MLE	
			UNAR	UNCS	UNAR	UNCS
Normal	3	3	0.77 (0.04)	0.80 (0.04)	0.65 (0.04)	0.66 (0.04)
		5	0.85 (0.04)	0.88 (0.04)	0.71 (0.04)	0.71 (0.04)
	5	3	0.85 (0.04)	0.89 (0.04)	0.66 (0.04)	0.66 (0.04)
		5	0.90 (0.03)	0.94 (0.03)	0.85 (0.03)	0.90 (0.02)
Poisson	3	3	0.93 (0.03)	0.94 (0.03)	0.78 (0.04)	0.79 (0.04)
		5	0.99 (0.01)	0.98 (0.03)	0.85 (0.05)	0.85 (0.05)
	5	3	0.99 (0.01)	0.99 (0.01)	0.90 (0.04)	0.92 (0.03)
		5	0.99 (0.01)	0.99 (0.01)	0.95 (0.02)	0.95 (0.02)
Mixed-type	3	3	0.74 (0.04)	0.75 (0.04)	0.56 (0.04)	0.55 (0.04)
		5	0.84 (0.04)	0.85 (0.04)	0.58 (0.04)	0.58 (0.06)
	5	3	0.83 (0.04)	0.86 (0.04)	0.58 (0.04)	0.58 (0.04)
		5	0.91 (0.03)	0.94 (0.03)	0.63 (0.04)	0.63 (0.04)

UNAR: unstructured between-responses and autoregressive order 1 within-response correlation matrix; UNCS: unstructured between-responses and compound symmetry within-response correlation matrix; GEE: generalized estimating equation; MLE: maximum likelihood estimation.

Moreover, RMDA based on GEE had the highest average classification accuracy compared to RMDA procedures based on MLE when responses were sampled from a multivariate Poisson distribution and mixed type responses. For example, when *P *=* *3 and *Q *=* *5, the average classification accuracies of RMDA procedures based on GEE and MLE were 0.97 and 0.84 when data were sampled from a multivariate Poisson distribution with response variables. Whereas, the average accuracy of the GEE and MLE procedures were 0.72 and 0.58, respectively, when mixed type responses, under UNAR correlation matrix (Table 3). In the quadratic discriminant analysis procedures, RMDA procedures based on MLE were least accurate regardless of number of repeated occasions, number of responses, estimation method or multivariate distribution of response variables (Table 4). For example, when Q = 5 and *P *=* *3 under UNAR correlation matrix, the average classification accuracies of RMDA procedures based on GEE and MLE were 0.85 and 0.66 when data were sampled from a multivariate normal distribution with response variables.

Furthermore, the average accuracy of each linear and quadratic discriminant analysis procedure increased as the number of repeated occasions and number of responses increased, regardless of the estimation method or multivariate distribution of response variables. For example, for Q = 3, when data were sampled from a multivariate normal distribution, the average increase in classification accuracy of the RMDA procedure based on GEE and MLE were about 0.11 and 0.05, respectively, as *p* increased from three to five, under UNCS correlation matrix (Table 3). Likewise, the average increase in classification accuracy of the RMDA procedure based on GEE and MLE were about 0.10 and 0.01, respectively, as *Q* increased from three to five, under UNCS correlation matrix and *P *=* *3 (Table 3).

It is worth mentioning that, we observed little or no differences in classification accuracies for linear and quadratic discriminant procedures when RMDA procedures based on MLE were used, whereas the classification accuracies for quadratic discriminant procedures based on GEE increased compared to its corresponding linear discriminant procedures (Tables 3 and 4)

For example: the average accuracy for RMDA procedure based on GEE and MLE were 0.64  and 0.65 , respectively, for linear discriminant procedure (Table 3), while for quadratic discriminant procedure, the average accuracies were 0.80 and 0.66, respectively (Table 4) under the UNCS correlation matrix, when data were sampled from a multivariate normal distribution with response variables and *P* = 3.

## 5 Health-Related Quality of Life in Children with Epilepsy Study (HERQULES)

Multivariate repeated measures data were obtained from the Health-Related Quality of Life (HRQOL) in Children with Epilepsy Study (HERQULES), a two-year prospective cohort study assessing the course and characteristics potentially associated with HRQOL in children with new onset epilepsy across Canada.^12,13^ Details of HERQULES have been described elsewhere.^12,13^ Data were collected as soon as possible following the diagnosis of epilepsy at baseline (0 month), and approximately 6 months, 12 months, and 24 months later. Standardized questionnaires were used to collect parent-report of their children’s HRQOL and a series of child and family characteristics, while a neurologist report form collected information on clinical characteristics of the child’s epilepsy.

Using these multivariate repeated measures data, we sought to identify patients who will not achieve remission from seizures within two years from disease onset. This group is referred to as the refractory group. A patient is defined as being in remission if they had a continuous 12-month period without any seizures at any point within two years from diagnosis.^3^ Early identification of patients who have refractory epilepsy can allow clinicians to explore alternative treatment options (e.g. surgery) to manage seizures and other aspects of the disease.^3^ Data for this numeric example consist of response variables such as severity of epilepsy,^14^ current number of anti-epileptic drugs (AEDs), and parent-reported quality of life in children using epilepsy-specific scale which were measured over four measurement occasions and the covariates were time of observation as a time-varying covariate, age at seizure onset, and sex as time invariant covariates. Repeated measures linear and quadratic discriminant analysis classification rules were developed based on multivariate GEE model using these data.

Of the 187 patients included in this analysis, 101 patients were in the remission group and 86 patients were in the refractory group within two years. The sample included children ages 4 to 12 years. The average age (standard deviation) in the remission group was 8.25 (2.46) years and in the refractory group was 8.25 (2.46) years. The patients included 45.54% and 41.86% females in the remission and refractory groups respectively. The QOLCE-55 ratings underwent a linear transformation such that domain scores yield values from 0 (low HRQOL) to 100 (high HRQOL). The ratings were treated as a continuous variable. The GASE scale is a seven-point Likert scale ranging from 1 (not severe at all) to 7 (extremely severe) was recoded as a binary variable, with ≥ 3 coded as severe, thereby using the median severity 3, corresponding to “somewhat severe” as a cut-off.^42^

### 5.1 Results for HERQULES data

**Figure 1.**
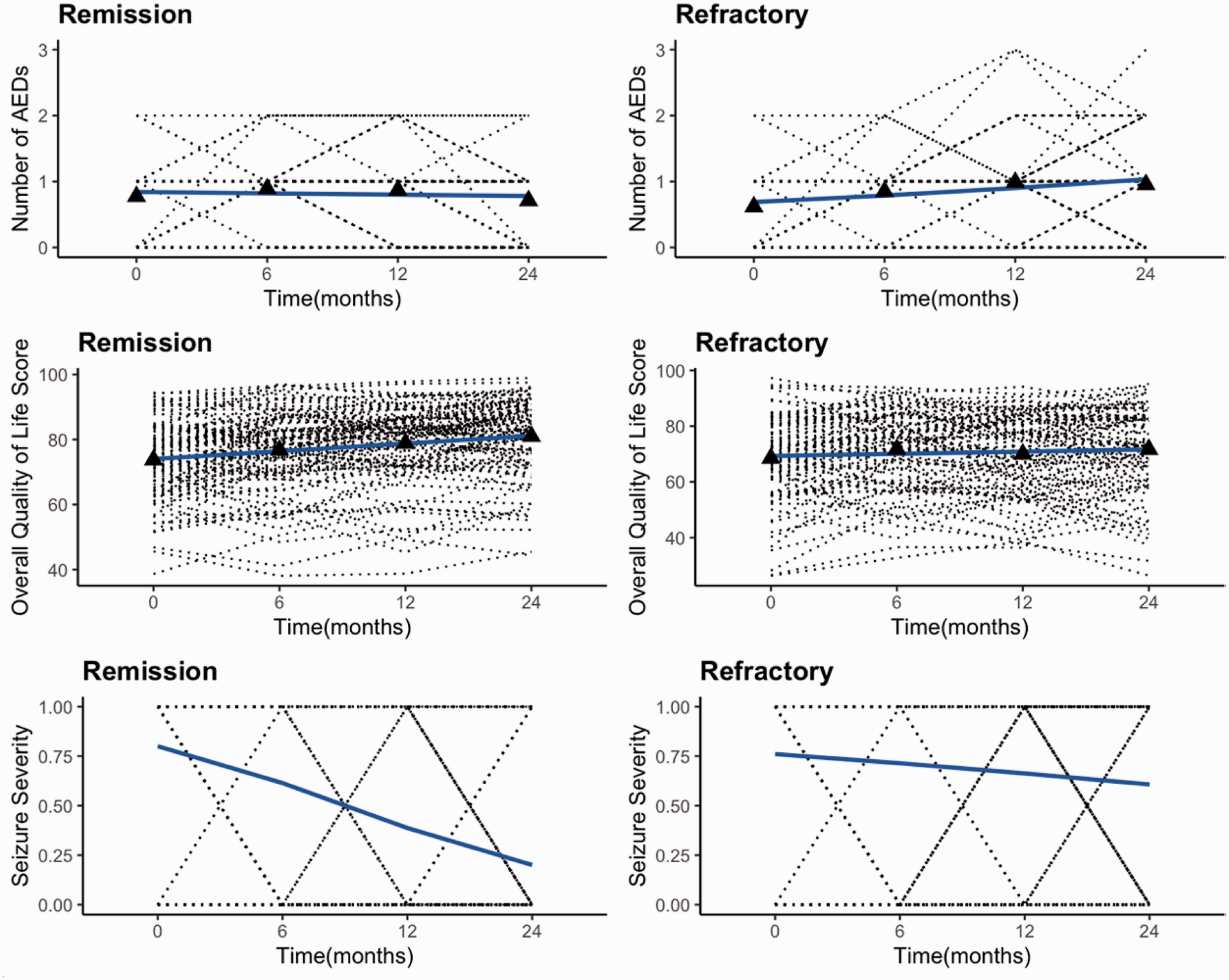
Observed longitudinal profiles of number of anti-epileptic drugs (AEDs), quality of life, and seizure severity from the Remission group (left column) and the Refractory group (right column). Solid lines show LOESS smoothed profiles for Poisson, normal, and binomial models calculated using data from all patients. Baseline (0 month), and 6 months, 12 months, and 24 months.

Figure 1 describes the longitudinal changes in the levels of each of the response variables for all patients in each diagnostic group. For patients who achieved remission, severity of seizures appears to decrease over time, whereas seizure severity remained high for the refractory group. The difference between the overall quality of life of the two groups is less noticeable. However, the overall quality of life appears constant over time in the refractory group, but as time increases, the overall quality of life of the remission patients gradually increases. The number of AEDs increased over time for the refractory patients, while those in the remission group had slightly reduced number of AEDs.

Table 5 gives the group-specific correlation parameter estimates of the joint modeling of the multiple repeated responses using multivariate GEE. We observed that in both remission and refractory groups, HRQOL was negatively associated with severity of seizures and the number of AEDs. However, there was little to no association between severity of seizures and the number of AEDs.

**Table 5.** GEE group-specific correlation parameter estimates for HERQULES data by the assumed correlation structure.

	Remission		Refractory	
	UNAR	UNCS	UNAR	UNCS
ρ	0.812	0.749	0.744	0.726
Corr(Y_2_Y_1_)	–0.025		–0.023	
Corr(Y_3_Y_1_)	0.003		0.001	
Corr(Y_3_Y_2_)	–0.042		–0.038	

UNAR: unstructured between-responses and autoregressive order 1 within-response correlation matrix; UNCS: unstructured between-responses and compound symmetry within-response correlation matrix; number of (AEDs) (**Y**_1_), HRQOL (**Y**_2_), Severe Seizure (**Y**_3_).

The accuracy of LDA and QDA classifiers based on GEE and maximum likelihood estimators are described in Table 6. Overall, RMDA procedures based on GEE exhibited higher overall classification accuracy than RMDA based on MLE in both LDA and QDA. Moreover, the classification accuracies observed using GEE estimators increased when QDA (accuracy, 0.79) was used for classification compared to its LDA (accuracy, 0.71) approach, while the accuracy using MLE estimators for remains the same for both QDA and LDA (accuracy, 0.67). The classifiers were more accurate in correctly reclassifying patients in the remission group but less accurate for reclassifying those in the refraction group.

**Table 6.** Classification accuracy for the generalized estimating equation (GEE), and maximum likelihood estimation (MLE) methods for repeated measures LDA and QDA by the assumed correlation structure.

	GEE		MLE	
	UNAR	UNCS	UNAR	UNCS
LDA
Remission	0.772	0.770	0.762	0.760
Refractory	0.651	0.640	0.570	0.558
Overall	0.711	0.705	0.665	0.660
QDA
Remission	0.871	0.880	0.752	0.750
Refractory	0.709	0.698	0.581	0.570
Overall	0.790	0.789	0.667	0.660

LDA: linear discriminant analysis; QDA: quadratic discriminant analysis; GEE: generalized estimating equation; MLE: maximum likelihood estimation; UNAR: unstructured between responses and autoregressive order 1 within response correlation matrix; UNCS: unstructured between responses and compound symmetry within response correlation matrix.

## 6 Discussion

This study investigated discriminant analysis procedures for multivariate repeated measures data using GEE for discriminating between population groups. The proposed approach allows the incorporation of repeated measures responses and covariates to improve the accuracy of the classifier. Our results showed that the RMDA based on GEE model resulted in better classification accuracy than the conventional RMDA based on maximum likelihood estimators especially in multivariate repeated measures data with discrete and/or mixed type of responses.^43,44^ This is because the GEE allows for the incorporation of multivariate repeated measures outcomes of different types without the need to fully specify the likelihood.^20,30,44,45^ Another advantage of these procedures is their ability to accommodate both time-invariant and time-varying covariate to improve the accuracy of modelclassifiers.

Furthermore, our study revealed the impact of increasing repeated occasions and number of responses on the accuracy of the investigated procedures. The impact of increasing number of repeated occasions is consistent with literature on other RMDA methods^22,37^; however, the studies from literature did not investigate the impact of increasing number of responses. Specifically, the RMDA based on GEE was most accurate with increases in the number of repeated occasions and number of responses compared to RMDA based on MLE. Overall, the quadratic discriminant analysis was able to better classify individuals than the linear discriminant analysis in RMDA based on GEE. QDA provides a less restrictive procedure by allowing different covariance matrixes for each group, which minimizes misclassification. Even though classification rules based on LDA can perform badly if the assumption of a common within-class covariance matrix is violated, classification rules based on QDA require a larger sample size to overcome the singularity problem.^13,46,47^ Even though the procedures developed in this study are based on two-group multivariate repeated designs, our conclusions can be extended and generalized to multi-group designs.^48,49^

Despite the unique strengths of this class of repeated measures discriminant analysis models, they are not without their limitations. First, the RMDA based on GEE relies on correctly specified link function and parsimonious covariance structures, which might not be tenable in typical multivariate repeated measures data. It is well known that GEEs yield asymptotically consistent parameter and variance estimates even under incorrect specification of the correlation structure but correctly specified link function.^18,43,45^ This means that a crucial step in the GEE approach is to select a correct link function linking the mean response to the covariates.^50^ With regard to parsimonious covariance structures, even though several authors have observed many advantages of using Kronecker product structure for analyzing multivariate repeated measures data,^22,24,37,51,52^ one could use the usual unstructured variance covariance matrix when there is sufficient data. Moreover, some work has been done on the testing of hypotheses of Kronecker product structure.^22,24,26^ It is also not clear whether the misspecification of the working correlation structures for these procedures could influence their classification accuracy.^53^ However, one does not know a priori which correlation structure is correct. Future research is needed to examine the impact of misspecification of covariance structure on the accuracy of these classifiers. In addition, to help in choosing a working correlation matrix that is close to the true correlation matrix, a quasi-likelihood under the independence model criterion (QIC) which is a modified Akaike information criterion (AIC) has recommended for GEE model.^54,55^ Secondly, the assumption of complete multivariate repeated measures data in which there are no missing data on all responses and at all measurement occasions might not be realistic in multivariate repeated measures data often encountered in applied research. Even in a well-controlled repeated measures study, missing data may frequently occur due to missed visits, withdrawal from the study, or loss to follow-up.^20^ Some studies have been done to address drop-out problems in repeated measures studies via weighted generalized estimating equations^56^ and imputations. Further research could extend the DA procedures based on GEE by implementing some of the multiple imputation techniques.^20,57–59^ Finally, our study focussed on comparing marginal models (GEE and covariance pattern models), which may not be efficient when accounting for individual-specific variations and dealing with missing data. Discriminant analysis based on mixed models constitute an alternative class of longitudinal classifiers that can account for individual-specific variations in longitudinal trajectories and accommodate incomplete longitudinal data, however, rely on the multivariate normality assumption.^7,9,15,16^ Future research will investigate the accuracy of discriminant analysis classifiers based on marginal and random-effects conditional models.

In summary, this study proposes a new class of discriminant analysis procedures based on GEE, which can be used for distinguishing between population groups in multivariate repeated measures data characterized by multivariate non-normal distributions with continuous, binary, or mixed types of response variables.
